# Synthesis of iron-based metal–organic framework@bacterial cellulose aerogels by an *in situ* growth method for multifunctional dye removal and oil–water separation applications

**DOI:** 10.1039/d5ra05250e

**Published:** 2025-09-18

**Authors:** Yang Chen, Shuhao Qin, Chengtao Gao, Xiao Wu, Min He, Daohai Zhang

**Affiliations:** a College of Materials and Metallurge, Guizhou University Guiang 550025 PR China; b National Engineering Research Center for Compounding and Modification of Polymer Materials, Guizhou Material Industrial Technology Institute Guiyang 550014 PR China pec.shqin@gzu.edu.cn; c School of Materials Science and Engineering, Guizhou Minzu University Guiyang 550025 PR China

## Abstract

Dyes and oily wastewater are among the most prevalent types of industrial wastewater pollutants, posing long-term threats to the environment and water resources due to their complex compositions and treatment difficulties. To achieve the separation and removal of these two categories of pollutants, this study developed two types of lightweight composite aerogels based on bacterial cellulose (BC). In this study, MIL-53-Fe (MIL-53) was loaded onto pure BC and polydopamine (PDA)-decorated BC (PBC) by *in situ* growth and then freeze-dried to prepare an MIL-53@BC aerogel (MIL-53@BCA) and MIL-53@PBC aerogel (MIL-53@PBCA), respectively. Results confirmed that both composite aerogels exhibited low densities (∼25 ± 5 mg cm^−3^) and robust structural stability. In terms of performance, both materials demonstrated promising potential for water treatment applications. Static adsorption experiments revealed that the equilibrium adsorption capacities of MIL-53@BCA and MIL-53@PBCA for Congo Red (CR) were 119.80 mg g^−1^ and 122.17 mg g^−1^, respectively. Adsorption kinetics conformed to the pseudo-first-order and pseudo-second-order models, while isotherm behavior was well-described by the Langmuir and Freundlich models. Thermodynamic analysis indicated that the adsorption process was spontaneous, exothermic, and entropy-increasing. Photocatalytic degradation experiments under visible light irradiation showed that both materials were effective in degrading methylene blue (MB) and rhodamine B (RhB). Gravity-driven oil–water separation testing confirmed the materials' ability to efficiently separate oil–water mixtures of varying densities and exhibited the simultaneous adsorption of residual dyes in the lower phase during the separation process. Therefore, the MOF-loaded cellulose aerogels developed in this study demonstrated integrated functionalities of adsorption, photocatalysis, and oil–water separation, proving their potential for the integrated remediation of wastewater treatment.

## Abstract 2

Dyes and oily wastewater are among the most prevalent types of industrial wastewater pollutants, posing longterm threats to the environment and water resources due to their complex compositions and treatment difficulties.

## Graphical abstract

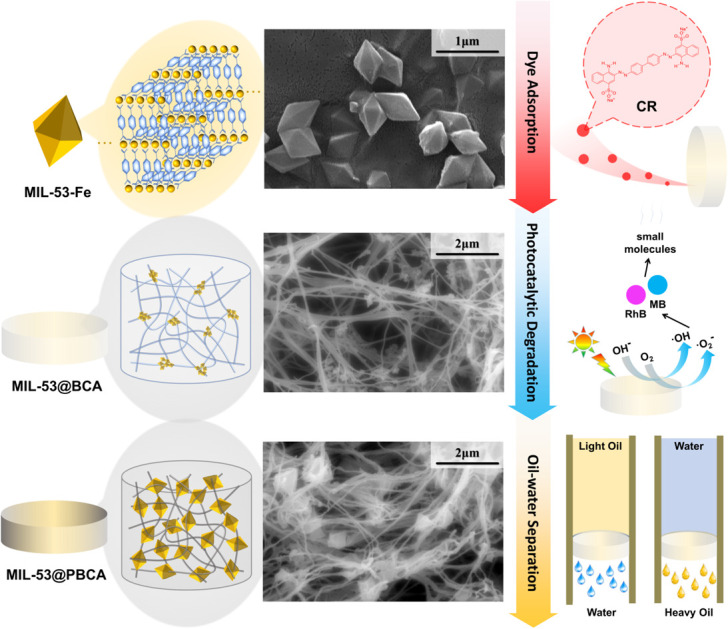

## 1 Introduction

Rapid industrial development has led to severe water pollution, characterized by dye and oily wastewater, which is difficult to treat due to the high stability and chemical complexity of dyes and oils.^1^ Traditional treatments, including flocculation, ion exchange, and biological methods, are often limited by inefficiency and the risk of secondary pollution.^2^ In response to these challenges, recent studies have focused on multifunctional materials, especially cellulose-derived aerogels for their renewability, high porosity, low density, and surface adaptability. The stable 3D network and abundant surface hydroxyl functionality of cellulose aerogels make them a great support matrix for metal–organic frameworks (MOFs) and hold great potential for treating complex wastewater.

MOFs are assembled by the coordination of metal nodes or organic ligands, offering tunable porous structures at the nano- and microscale levels.^3^ MOFs also offer tunable shapes and chemical characteristics for specific application needs.^4^ The large surface area and chemical flexibility of MOFs exhibit strong potential in gas capture, contaminant removal, photocatalysis, and resource reutilization.^5–9^ However, MOFs face several practical limitations, including water-induced degradation, the potential toxicity of ligands, high production costs, challenges in mass production, and poor recyclability.^10,11^ To address these challenges, MOFs can be anchored onto mechanically stable and structurally tunable substrates, thereby improving their usability in practical cases. As typical scaffolds, cellulose-derived aerogels, owing to their sustainability, eco-friendliness, and inherent ability to stabilize and evenly distribute MOFs, are ideal for hybrid material design.^12,13^ The high surface functionality of cellulose can not only control MOF crystallization but also play a key role in maintaining its framework integrity and accessible pore structure. Therefore, MOF@cellulose aerogels are of great interest as multifunctional platforms for water purification applications.

Previous work has demonstrated that MOFs like UiO-66,^14,15^ MIL-101-Fe,^16^ ZIF-8,^17^ and CuBDC^18^ with cellulose scaffolds can improve the adsorption or photocatalytic capabilities of pollutants, including dye molecules and heavy metal ions. The functionalized MOF@cellulose exhibits superwetting behavior, enabling gravity-driven oil–water separation in multifunctional water purification applications. However, current studies are limited to achieving only one or two functionalities, normally adsorption or photocatalysis, while the integrated systems for simultaneously performing adsorption, photocatalytic degradation, and oil–water separation remain rare. Therefore, to bridge this gap, this work proposes to develop multifunctional composite aerogels based on bacterial cellulose (BC) as a robust framework. Bacterial cellulose (BC) was initially decorated with a polydopamine (PDA) layer to enhance interfacial compatibility, followed by the *in situ* assembly of MIL-53-Fe (MIL-53, an iron-based metal–organic framework) to generate a hierarchically porous framework. The resulting MIL-53@cellulose aerogel was fully characterized, and its multifunctional capabilities, including dye adsorption, photocatalytic activity, and oil–water separation under gravitational drive, were systematically examined. This strategy offers a solution for treating dyes and oils from wastewater, highlighting the aerogel's potential for sustainable and biodegradable applications in environmental remediation.

## 2 Experimental

### 2.1 Materials

The bacterial strain *Acetobacter xylinum* ATCC 23767 was purchased from the Beijing Microbiological Culture Collection Center. Yeast extract was purchased from OXOID. Peptone was purchased from Beijing Aoboxing Biotechnology. d-glucose anhydrous (AR), citric acid (AR), magnesium sulfate (MgSO_4_, AR), sodium dihydrogen phosphate (NaH_2_PO_4_, AR), sodium hydroxide (NaOH, AR), hydrochloric acid (HCl, 37%), ferric chloride hexahydrate (FeCl_3_·6H_2_O, AR), terephthalic acid (TPA, AR), *N*,*N*-dimethylformamide (DMF, AR), hydrogen peroxide (H_2_O_2_, 30%), glacial acetic acid (AR), tetrachloromethane (CCl_4_, AR) and ethanol (AR) were purchased from Shanghai Macklin Biochemical Co. Ltd. Dopamine hydrochloride (99.5%) and Tris-hydrochloride buffer (Tris–HCl, 10 mM, pH = 8.5) were purchased from Shanghai Macklin Biochemical Co. Ltd. Congo Red (CR, 99%), methylene blue (MB, 99%), rhodamine B (RhB, 99%) and methyl red (99%) were purchased from Shanghai Macklin Biochemical Co. Ltd. All chemicals were used as received without any purification.

### 2.2 Production of bacterial cellulose (BC)

Bacterial cellulose (BC) aerogels were prepared following the steps below. Briefly, a nutrient medium was prepared by dissolving 40.0 g of d-glucose, 5.0 g of yeast extract, 5.0 g of peptone, 2.7 g of NaH_2_PO_4_, 1.4 g of citric acid, 0.6 g MgSO_4_, and 10.0 mL ethanol in 900 mL of deionized water under stirring. The solution was topped up to 1000 mL with deionized water, and then the pH was adjusted to 5.5 by the gradual dripping of glacial acetic acid. After cultivating with *Acetobacter xylinum* ATCC23767, the culture was first incubated under shaking conditions at 30 °C (180 rpm) to promote bacterial activation and initial cellulose floc formation. This was followed by static incubation at the same temperature for several days, after which a uniform BC membrane (∼0.5 cm thick) formed at the air–liquid interface.

### 2.3 Synthesis of bacterial cellulose aerogel (BCA)

The as-acquired bacterial cellulose pellicle was immersed in 1.0 mol L^−1^ NaOH solution and purified at 85 °C under continuous stirring for 2 hours to remove medium components. The membranes were then washed with deionized water until a neutral pH was reached. Subsequently, the purified bacterial cellulose was subjected to freeze-drying at −54 °C until its weight became constant. The resulting lightweight, porous material was designated as the BCA substrate and stored for further modification and characterization.

### 2.4 Synthesis of polydopamine-coated bacterial cellulose aerogel (PBCA)

Here, 40 mg of dopamine hydrochloride (DA HCl) was fully dissolved in Tris buffer solution under light-excluded conditions, and then the BCA was fully immersed in the DA solution for 24 hours at room temperature, enabling the self-polymerization of dopamine to create a PDA layer on the aerogel surface. After incubation, the resulting PDA-coated sample was rinsed with warm water (∼35 °C) to remove loosely attached PDA residues, followed by thorough washing with deionized water until a neutral pH was achieved. The cleaned material was then freeze-dried at −54 °C until completely dried, yielding the PBCA substrate for subsequent use.

### 2.5 Synthesis of MIL-53-Fe (MIL-53)

**Fig. 1:**
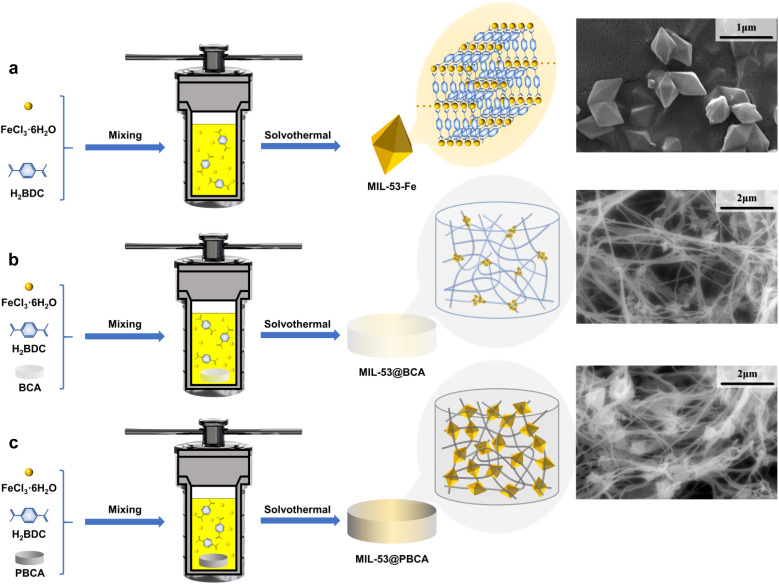
Schematic of the *in situ* preparation of (a) MIL-53, (b) MIL-53@BCA and (c) MIL-53@PBCA.

Here, 1.35 g of FeCl_3_·6H_2_O and 0.83 g of terephthalic acid (TPA) were dissolved in 25 mL of DMF at 35 °C, and then transferred to a 100 mL Teflon autoclave, where the temperature was raised to 152 °C and kept for 24 h to obtain the pure MIL-53 metal–organic framework crude product. The raw powder was ultrasonically washed with DMF, ethanol, and deionized water at 35 °C until the excess chemicals were removed, and then dried under vacuum at 120 °C to obtain the final MIL-53 nanoparticles. The synthesis process of MIL-53 is illustrated in Fig. 1a.

### 2.6 Synthesis of MIL-53@BCA and MIL-53@PBCA

To synthesize MIL-53-loaded BCA and PBCA, 1.35 g of ferric chloride hexahydrate (FeCl_3_·6H_2_O) and 0.83 g of terephthalic acid (TPA) were dissolved in 25 mL of *N*,*N*-dimethylformamide (DMF) at 35 °C under constant stirring. The as-synthesized BCA and PBCA substrates were fully immersed in the precursor solution and transferred into a 100 mL Teflon autoclave. The sealed reactor was then heated to 152 °C and maintained statically for 24 hours. After the reaction, the resulting MIL-53@BCA and MIL-53@PBCA composites were retrieved and subjected to repeated ultrasonic washing with DMF, ethanol, and deionized water at 35 °C to remove unreacted chemicals. Finally, the cleaned aerogels were dried under vacuum at 120 °C until completely dehydrated, yielding MIL-53-loaded composite aerogels for subsequent characterization and application. Fig. 1b and c is the Schematic illustration of the fabrication process for the MIL-53@BCA and MIL-53@PBCA composite aerogels.

### 2.7 Characterization

The microstructural features of the aerogel samples were examined using scanning electron microscopy (SEM), allowing the direct observation of their porous morphology. Before crystal structure analysis, samples were cryogenically fractured in liquid nitrogen and ground into fine powders. Powder X-ray diffraction (XRD) was then conducted using a Bruker AXS D8 Advance diffractometer equipped with Cu Kα radiation, scanning over a 2*θ* range of 5° to 80° to identify crystalline phases. To investigate the chemical functionalities, Fourier-transform infrared spectroscopy (FTIR) was carried out using a Thermo Scientific NICOLET iS50 instrument equipped with an attenuated total reflectance (ATR) accessory. UV-vis diffuse reflectance spectra (UV-vis DRS) were obtained using a Jasco V-550 UV-vvis spectrophotometer with BaSO_4_ as a reflectance standard. Thermal stability and decomposition behavior were evaluated *via* thermogravimetric analysis (TGA) using a HITACHI STA200 TGA 8000 analyzer. Measurements were carried out under a nitrogen atmosphere (flow rate: 40 mL min^−1^) with a heating rate of 5 °C min^−1^ from room temperature up to 792 °C. The following equation^18–21^ was applied to calculate the loading content of MIL-53 on both MIL-53@BCA and MIL-53@PBCA:1
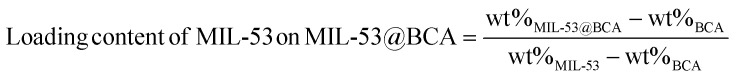
2
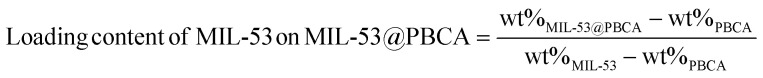
where wt%_MIL-53@BCA_, wt%_MIL-53@PBCA_, wt%_BCA_, wt%_PBCA_, and wt%_MIL-53_ represent the remaining weight percentages of MIL-53@BCA, MIL-53@PBCA, pure BCA, pure PBCA and pure MIL-53, respectively, at the final temperature of 792 °C.

### 2.8 Adsorption, photocatalytic degradation, and gravity-driven oil–water separation performance

Congo Red (CR) was chosen to evaluate the adsorptive performance of the MIL-53@BCA and MIL-53@PBCA composite. Firstly, standard CR solutions in different concentrations ranging from 100 mg L^−1^ to 300 mg L^−1^ were prepared. Then, 10 mg of the two as-prepared aerogels were added to 50 mL of the dye solution at pH = 7.0, respectively. For the measurements, the concentrations of CR solution at different time intervals and equilibrium were detected by an ultraviolet-visible spectrophotometer (Ultrospec 6300, Cytiva, UK) at a wavelength of 498 nm. The equilibrium adsorption capacity (*Q*_e_) and adsorption capacity at different time points (*Q*_*t*_) of CR are as follows:3
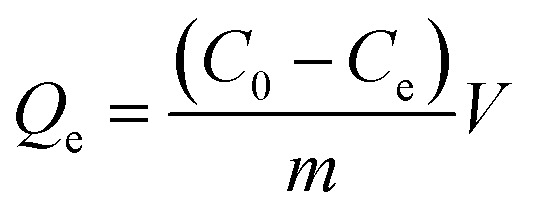
4
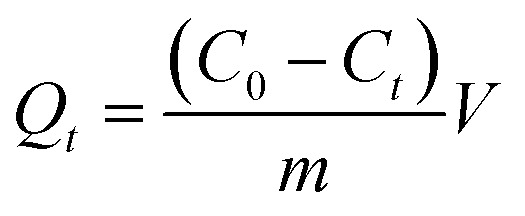
where *Q*_e_ (mg g^−1^) is the equilibrium adsorption capacity of each sample; *Q*_*t*_ (mg g^−1^) is the adsorption capacity at different time points; *C*_0_ (mg L^−1^) is the initial dye concentration, *C*_e_ (mg L^−1^) is the equilibrium concentration and *C*_*t*_ (mg L^−1^) is the current concentration at different time points; *V* (mL) is the volume of solution, and *m* (mg) is the sample weight.

The adsorption kinetics were fitted with both pseudo-first-order (PFO) and pseudo-second-order (PSO) models,^22–25^ which are presented as follows:

5 *Q*_*t*_ = *Q*_e_(1 − *e*^−*k*_1_*t*^)

6
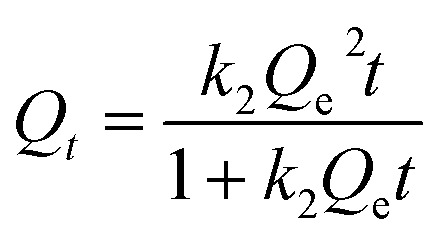
where *Q*_e_ (mg g^−1^) is the equilibrium adsorption capacity; *Q*_*t*_ (mg g^−1^) is the adsorption capacity at time *t*; *k*_1_ (min^−1^) and *k*_2_ (g mg^−1^ min^−1^) are the rate constants of PFO and PSO, respectively.

The adsorption isotherm experiment was carried out at temperatures of 303, 313, and 323 K, and the data were fitted using the Langmuir and Freundlich isotherm models,^26,27^ which are expressed as follows:7
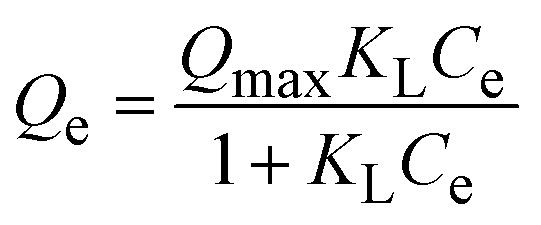

8 *Q*_e_ = *K*_F_ × *C*^1/*n*^_e_

where, *K*_L_ (L mg^−1^) is the Langmuir constant and *Q*_max_ (mg g^−1^) is the maximum adsorption capacity of the theoretical monolayer; *K*_F_ (mg^1−1/*n*^ L^1/*n*^ g^−1^) is the Freundlich constant and *n* is the heterogeneity factor, which reflects the degree of irregularity of the adsorbant surface.

The thermodynamic parameters: Gibbs free energy (Δ*G*°), enthalpy (Δ*H*°), and entropy (Δ*S*°) of adsorption thermodynamics were calculated using the Van't Hoff equation,^28,29^ and are expressed as follows:

9 Δ*G*° = −*RT* ln *K*_L_

10
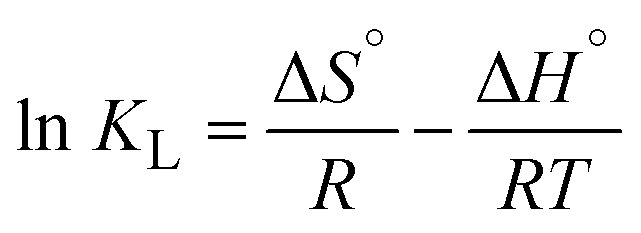
where *R* is the gas constant (8.314 J mol^−1^ K^−1^), *T* is the temperature (K), and *K*_L_ is the Langmuir adsorption constant (L mol^−1^) calculated from the Langmuir isotherm models.

The cycle testing of the MIL-53@BCA and MIL-53@PBCA composite aerogels for adsorbing CR was conducted for 5 cycles. After each cycle, the aerogels were immersed in ethanol to extract CR until the liquid phase was totally transparent, and fully dried until no obvious mass was lost. Then, a repeat experiment was conducted for the next adsorption.

CR was reserved for adsorption studies rather than photocatalysis because both MIL-53@BCA and MIL-53@PBCA exhibit a strong affinity toward anionic CR, which would confound the kinetics attribution between adsorption and photo-degradation. In contrast, methylene blue (MB) and rhodamine B (RhB) showed negligible uptake after the 60 minutes dark equilibration (methods), enabling clean assessment of visible-light/H_2_O_2_-driven photocatalysis without adsorption interference. Therefore, to evaluate the photocatalytic activity of the MIL-53@BCA and MIL-53@PBCA composites, MB and RhB were selected as representative organic dye pollutants. Simulated solar irradiation was provided by a 300 W xenon lamp. For each test, 5.0 mg of the aerogel composite was dispersed into 20 mL of dye solution (initial concentration: 100 mg g^−1^, pH = 7), and the mixture was magnetically stirred at 50 rpm in the absence of light for 60 minutes to allow adsorption–desorption equilibrium to be established. Following this dark equilibration step, hydrogen peroxide (H_2_O_2_, 6 mL g^−1^) was introduced as a reactive oxygen species promoter under continuous light exposure. Aliquots were collected at designated intervals, appropriately diluted, and analyzed using a UV-vis spectrophotometer (Ultrospec 6300, Cytiva, UK). The absorbance was monitored at 664 nm for MB and 554 nm for RhB. In addition to qualitative assessments, the photocatalytic degradation behavior was quantitatively investigated using kinetic modeling. The degradation profiles were fitted to a pseudo-first-order model to gain insight into the reaction dynamics by the equation as follows:^30,31^11
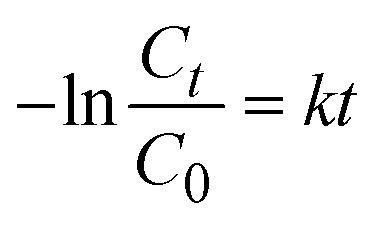
where *C*_0_ (mg L^−1^) is the initial dye concentration and *C*_*t*_ (mg L^−1^) is the current concentration at time *t*; *k* is the reaction constant.

The cycle test was carried out for 5 cycles. After each cycle, the aerogels were washed with DIW and ethanol, and fully dried in an oven until no obvious mass was lost. Then, the experiment was repeated for the next photocatalytic degradation.

To assess the oil/water separation performance, the two as-prepared aerogels were compressed and securely positioned at the center of a custom-built gravity-driven filtration apparatus. Disk-shaped aerogels were cut to 3.0 mm thickness (diameter matching the holder), pre-wetted with the intended permeating lower phase to soften and set the wettability, and then uniformly compressed to ∼1.0 ± 0.1 mm within the clamp before loading into the apparatus. After compression, the bottom valve was closed to initiate the observation period described above. Two representative biphasic systems were tested: an *n*-hexane/water mixture representing light oil–water separation, and a water/carbon tetrachloride (CCl_4_) mixture mimicking heavy oil–water conditions. Each biphasic system was prepared by mixing equal volumes of oil and water (15 mL : 15 mL). In the light oil system, the aqueous phase was dyed with Congo Red (10 mg L^−1^) for visualization, and the aerogel was pre-wetted with water to ensure preferential water permeability. Upon pouring the mixture into the upper compartment of the filtration setup, a clear stratification was observed. As the separation proceeded under gravity, the hydrophilic aerogel selectively allowed water to pass through while effectively repelling the lighter *n*-hexane, which remained in the upper chamber. After full drainage of the water phase, *n*-hexane was collected from the upper reservoir, and the aqueous filtrate was collected below. For the heavy oil–water system, the procedure was similarly conducted, with minor adjustments. CCl_4_ was used as the heavy oil phase and dyed with methyl red (10 mg L^−1^), while the aerogel was preconditioned with CCl_4_ to reverse the wettability preference. In this case, the denser CCl_4_ permeated the aerogel and was collected from the bottom chamber, whereas the water phase remained above. To quantify the separation performance, the passage time of the permeable (lower) phase through the aerogel was recorded. Additionally, after complete permeation of the lower phase, the bottom stopcock was closed, and the assembly was kept static. Effective blocking was defined as no droplet breakthrough, meniscus movement, or change in phase boundary at the aerogel interface during a ≥360 min observation. These criteria were applied to both light-oil (*n*-hexane/water) and heavy-oil (CCl_4_/water) systems.

## 3 Results and discussions

### 3.1 Characterizations

**Fig. 2:**
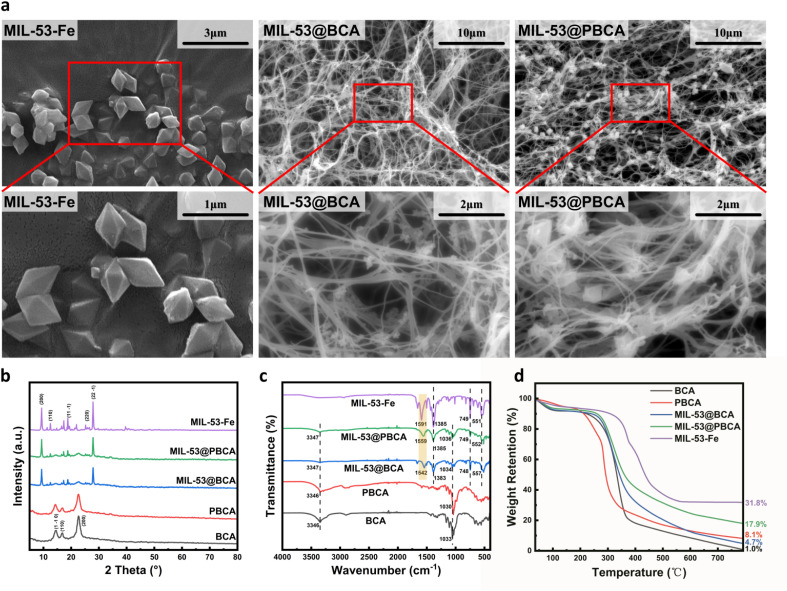
(a) SEM images of MIL-53, MIL-53@BCA, and MIL-53@PBCA; (b) XRD patterns, (c) FTIR spectra, and (d) TGA curves of MIL-53, BCA, PBCA, MIL-53@BCA and MIL-53@PBCA composite aerogels.

As shown in Fig. 2a, the BCA aerogel displays a clearly defined, highly porous, and entangled fibrous network, while with PDA coating, the PBCA sample shows no notable morphological changes compared to BCA. The fiber surfaces remain similarly textured, indicating that the PDA layer does not significantly alter the surface smoothness or network architecture at the microscale. In contrast, the incorporation of MIL-53 into the aerogels results in more distinct differences, as illustrated in MIL-53@BCA and MIL-53@PBCA in Fig. 1b. For MIL-53@BCA, the distribution of MOF particles is not obvious in the low-magnification image; only upon local magnification can small-sized MIL-53 crystals be observed, slightly anchored on the cellulose network. This limited visibility is likely due to the relatively fine, fibrillar structure of BCA, which provides less spatial accommodation for crystal growth, resulting in smaller and less MOF deposits – especially when compared to larger-scale substrates such as microcrystalline cellulose discussed in earlier sections. However, MIL-53@PBCA exhibits a much clearer and more uniform MOF distribution across the fibrous matrix. The PDA coating appears to enhance nucleation density by introducing additional –OH and –NH_2_ functional groups, offering more active coordination sites for Fe^3+^. As a result, MIL-53 crystallites preferentially form at junctions or entanglement points within the network, giving rise to a more stable “crosslinked” structure that integrates well with the aerogel framework.

Fig. 2b shows the X-ray diffraction (XRD) patterns of MIL-53 powder, BCA, PBCA, MIL-53@BCA, and MIL-53@PBCA. Both BCA and PBCA samples exhibit three typical diffraction peaks at approximately 14.8°, 17.1°, and 23.0°, corresponding to the (1 1 0), (1 1 0), and (2 0 0) planes of cellulose I, respectively.^32–34^ These peaks confirm the preservation of the native crystalline structure of BC in both untreated and PDA-modified aerogels. The XRD pattern of PBCA remains nearly identical to that of BCA, indicating that the polydopamine coating does not significantly alter the crystalline structure of the cellulose matrix. In comparison, the pure MIL-53 powder displays characteristic reflections at 9.34°, 12.50°, 17.35°, 18.77°, 21.92°, and 27.88°, which are consistent with the known crystal structure of MIL-53.^35^ For both MIL-53@BCA and MIL-53@PBCA composites, the XRD spectra clearly reveal the coexistence of diffraction peaks associated with MIL-53 and those of the original cellulose aerogel. This observation confirms the successful integration of MOF crystals into the cellulose framework. Notably, the presence of well-resolved MIL-53 peaks in the composites suggests that the links, involving hydrogen bonding or electrostatic interactions, did not disrupt the original crystalline integrity of MIL-53. These structural features are in agreement with SEM observations, which similarly showed clear and integrated crystal morphologies anchored within the aerogel networks.

Fourier-transform infrared (FTIR) spectroscopy was carried out to show the interactions between MIL-53 and the BCA/PBCA substrates. As shown in Fig. 2c, the FTIR spectrum of BCA exhibits characteristic cellulose absorption bands at 3346 cm^−1^ and 1033 cm^−1^, corresponding to O–H stretching and C–O/C–C stretching vibrations, respectively.^27^ After the *in situ* growth of MIL-53, new absorption bands appeared in the composite spectra, including peaks associated with terephthalate (TPA) groups at 1685, 1542, 1383, and 748 cm^−1^, along with a Fe–O stretching vibration at 557 cm^−1^, confirming the successful linkage of MIL-53 onto the aerogel matrix.^27^ A similar spectral pattern was observed in the PBCA sample, with peaks at 3346 cm^−1^ and 1030 cm^−1^ indicating retained –OH and C–O/C–C functionalities. The MIL-53@PBCA spectrum displays characteristic signals from both MIL-53 and the PBCA substrate, further verifying that MIL-53 was also successfully grown on the PDA-modified cellulose surface. This supports the hypothesis that hydroxyl and amine groups present on the BCA and PBCA frameworks serve as active nucleation sites, enabling coordination with Fe^3+^ ions and facilitating *in situ* crystal growth *via* hydrogen bonding and electrostatic interactions. The asymmetric stretching band of the carboxylate group, typically located at 1591 cm^−1^ in pure MIL-53, exhibits a shift to 1542 cm^−1^ in MIL-53@BCA and to 1559 cm^−1^ in MIL-53@PBCA. This shift suggests that the coordination environment of TPA during MOF formation is influenced by the surface of the substrate. In particular, differences in nucleation density and local chemical environments, especially the presence of abundant activated sites in PBCA, may contribute to the variation in crystal size and growth behavior observed in the composites compared to the pristine MIL-53 synthesized in solution.

The thermogravimetric analysis (TGA) curves of MIL-53 powder, BCA, PBCA, MIL-53@BCA, and MIL-53@PBCA are displayed in Fig. 2d. Both BCA and PBCA exhibit significant mass loss between 300 °C and 350 °C, which is attributed to the thermal degradation of cellulose glycosidic linkages under increasing temperatures. The TGA profile of MIL-53 reveals two distinct stages of weight loss. The initial loss observed between 50 °C and 200 °C corresponds to the removal of adsorbed water and DMF solvent. More substantial mass loss occurs between 300 °C and 500 °C due to the thermal collapse of the TPA-based MOF framework,^36^ which is not stable at higher temperatures. This breakdown is accompanied by the oxidation of Fe^3+^ species into iron oxides.^27^ For both MIL-53@BCA and MIL-53@PBCA, the thermal degradation behaviors are similar to those of their respective aerogel matrices. Based on calculations derived from eqn (1) and (2) in Section 2.7, the MIL-53 loading contents in MIL-53@BCA and MIL-53@PBCA are estimated to be 12.1% and 41.2%, respectively. The considerable difference in MOF loading can be ascribed to the underlying structural characteristics of the cellulose substrates. In the BCA-based composite, MIL-53 tends to form smaller particles with limited spatial anchoring, as the fiber network offers few robust binding sites for crystal growth. In contrast, PBCA provides a more favorable environment for MOF deposition. The presence of PDA introduces additional functional groups that perform stronger interactions between MIL-53 crystals and the cellulose framework. As a result, larger and more uniformly distributed MIL-53 particles are observed, leading to higher MOF content. Furthermore, the relatively low density of the bacterial cellulose network, coupled with dense and large-sized MOF crystallites, leads to the high loading ratio of MOF in the PBCA system.

### 3.2 Adsorption performance analysis

#### 3.2.1 Adsorption kinetics

**Fig. 3:**
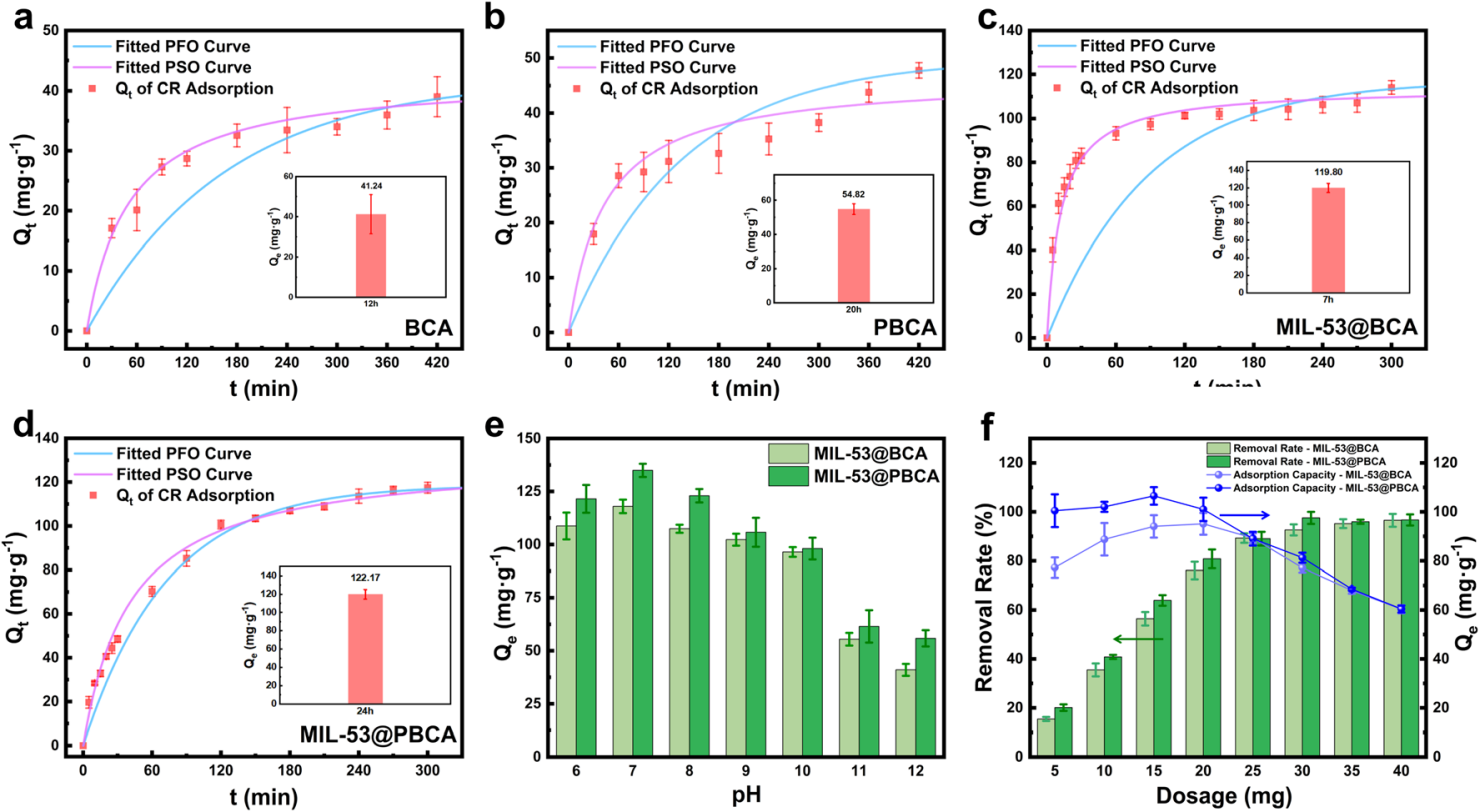
PFO and PSO kinetics of (a) BCA, (b) PBCA, (c) MIL-53@BCA, and (d) MIL-53@PBCA for CR adsorption; equilibrium adsorption capacity (for CR) of MIL-53@BCA and MIL-53@PBCA in different (e) pH, and (f) doses of the adsorbent.

Fig. 3a–d shows the adsorption behaviors of Congo Red (CR) on MIL-53@BCA and MIL-53@PBCA composite aerogels, in comparison with pristine BCA and PBCA. Experimental results indicate that both unmodified aerogels exhibit comparatively low adsorption capacities. Specifically, BCA reached equilibrium after 12 hours with a maximum adsorption capacity of 41.24 mg g^−1^, while PBCA required 20 hours to reach 54.82 mg g^−1^. The delayed equilibrium in PBCA suggests that the introduction of polydopamine (PDA) did not significantly enhance the adsorption kinetics and may have slightly reduced the initial adsorption rate. This behavior is likely related to changes in surface chemistry. In BCA, the native entangled fiber network exposes many hydroxyl groups that can interact with dye molecules, facilitating faster adsorption. Upon PDA modification, the surface becomes enriched with amino groups and π-conjugated structures. However, these functionalities may exhibit weaker or less rapid interactions toward CR than hydroxyl groups, resulting in a slower approach to equilibrium. Despite this, the rougher surface texture and high density of functional groups (*e.g.*, –OH, –NH_2_) in PBCA contribute to a higher overall adsorption capacity than unmodified BCA. Kinetic parameters further elucidate the adsorption mechanisms. As shown in Table 1, the adsorption data for BCA fit better with the pseudo-second-order (PSO) model than the pseudo-first-order (PFO) model, indicating a dominant chemisorption process involving specific interactions between CR and the cellulose surface. Although BCA's high porosity may play a major role in dye diffusion, the contribution from physical adsorption appears limited. In contrast, PBCA exhibits high correlation coefficients (*R*^2^ > 0.950) for both PFO and PSO models, suggesting that its adsorption involves a synergistic mechanism with both physical diffusion and chemical binding at functional sites. The presence of a dense distribution of active groups on PBCA likely promotes both internal diffusion and surface–level interactions, resulting in enhanced overall dye removal performance.

**Table 1:** Adsorption kinetics parameters of BCA, PBCA, MIL-53@BCA and MIL-53@PBCA

	Pseudo first order			Pseudo second order		
	*Q*_e_ (mg g^−1^)	*k*_1_	*R* ^2^	*Q*_e_ (mg g^−1^)	*k*_2_	*R* ^2^
Pure BCA	39.36	0.00302	0.925	39.76	4.680 × 10^−4^	0.992
Pure PBCA	52.77	0.00438	0.954	52.80	2.631 × 10^−4^	0.959
MIL-53@BCA	107.29	0.01132	0.891	112.10	7.293 × 10^−4^	0.997
MIL-53@PBCA	108.69	0.01376	0.978	131.93	1.819 × 10^−4^	0.994

The decoration of MIL-53 can significantly enhance the adsorption capacity of the aerogels toward Congo Red (CR), although its influence on adsorption kinetics differed between the two composite systems. As shown in Fig. 2c and d, MIL-53@BCA exhibited a rapid initial adsorption rate followed by a notable decrease in the later stages, eventually reaching equilibrium within a short period. This behavior can be attributed to the abundant active sites provided by the finely dispersed MIL-53 nanocrystals on the BCA surface, which enable fast chemisorption of CR molecules in the early phase.^37–39^ However, the spatial limits of the BCA fibers restrict sustained dye accumulation, thereby capping the overall adsorption capacity. In contrast, MIL-53@PBCA exhibited a gradual increase in adsorption capacity, ultimately reaching 131.93 mg g^−1^ as calculated by the pseudo-second-order kinetic model. The presence of larger MIL-53 crystals, more uniformly distributed in the PDA-modified network, prolonged dye capture *via* specific interactions such as coordination bonding and surface complexation. Kinetic parameters derived from Table 1 also show that, regardless of MIL-53 loading, the introduction of PDA enhanced the initial diffusion-driven adsorption, as evidenced by the increased pseudo-first-order rate constant (*k*_1_) in PBCA relative to BCA, and in MIL-53@PBCA relative to MIL-53@BCA. This improvement may be ascribed to the increased surface roughness and functional group density imparted by the PDA layer, which accelerates early-stage dye penetration. For the pseudo-second-order model, MIL-53 loading led to an increase in the *k*_2_ value for MIL-53@BCA, reflecting faster chemical adsorption and higher equilibrium capacity, albeit with rapid saturation. On the other hand, the adsorption process in MIL-53@PBCA was characterized by a slower progression toward equilibrium despite reaching a higher ultimate capacity. This delay can be attributed to the interplay of multiple binding interactions – such as electrostatic attraction, π–π stacking, and hydrogen bonding between CR molecules and the densely functionalized MOF–polymer interface. The presence of more active sites likely promoted sustained adsorption but extended the time required to achieve full occupation, resulting in a lower *k*_2_ value compared to its BCA-based counterpart.

#### 3.2.2 The effects of pH and dosage of absorbent on the adsorption

Fig. 3e compares the equilibrium adsorption capacities of MIL-53@BCA and MIL-53@PBCA composite aerogels toward Congo Red (CR) under varying pH conditions and different adsorbent dosages. As shown in Fig. 3e, both materials exhibit optimal adsorption performance at around pH = 7. This enhancement can be attributed to the electrostatic interactions between the weak adsorbent surface and the anionic dye molecules. Near-neutral pH allows the surface of the composites to carry slight positive charges, which effectively attract the negatively charged sulfonate groups in CR, thereby promoting adsorption.^40^ However, as the solution becomes more alkaline, the adsorption capacity declines. This decrease is primarily caused by the increasing negative surface potential of the adsorbents, which repels the anionic dye molecules and suppresses other interactions such as hydrogen bonding.^41^ Additionally, the presence of excess hydroxide ions (OH^−^) under basic conditions may competitively occupy active adsorption sites, further inhibiting dye uptake.^42^ Once the pH exceeds 10, functional groups such as hydroxyl (–OH) and carboxyl (–COOH) on the aerogel surface undergo deprotonation, forming stable –O^−^ and –COO^−^ species. These negatively charged moieties intensify the electrostatic repulsion with CR, leading to a more pronounced reduction in adsorption efficiency.^40^ These observations indicate that both MIL-53@BCA and MIL-53@PBCA perform best under mildly acidic to neutral conditions (pH = 7), while their adsorption capacities decrease under strongly alkaline or weakly acidic environments.

Fig. 3f illustrates the adsorption behavior of MIL-53@BCA and MIL-53@PBCA composite aerogels toward Congo Red (CR) under varying dosages of adsorbent. The data reveal a general trend as follows: as the amount of aerogel increases, the overall removal efficiency of CR improves; however, the adsorption capacity per unit mass of adsorbent (*Q*_e_) initially rises to a maximum and then declines with further dosage increases.^40^ This pattern can be attributed to the interplay between adsorbent availability and dye molecule accessibility. At lower dosages, the ratio of dye molecules to available adsorption sites is relatively high, allowing each active site to be efficiently utilized. As the dosage increases, more sites become available, and the total amount of adsorbed dye rises accordingly. However, beyond a certain threshold, the excess adsorbent no longer leads to proportional gains in dye removal because the fixed quantity of dye becomes insufficient to saturate the increasing number of available sites. Consequently, the average adsorption capacity per gram of material diminishes due to site underutilization.^41^ These findings underscore the importance of dosage optimization in practical wastewater treatment applications. The overuse of adsorbents not only reduces material efficiency but also increases treatment costs unnecessarily. Therefore, determining an appropriate adsorbent-to-pollutant ratio is critical to achieving both high removal performance and economic viability. A well-balanced dosing strategy ensures that the adsorption capacity of the material is effectively harnessed while maintaining cost efficiency in large-scale operations.

#### 3.2.3 Adsorption isotherm models

**Fig. 4:**
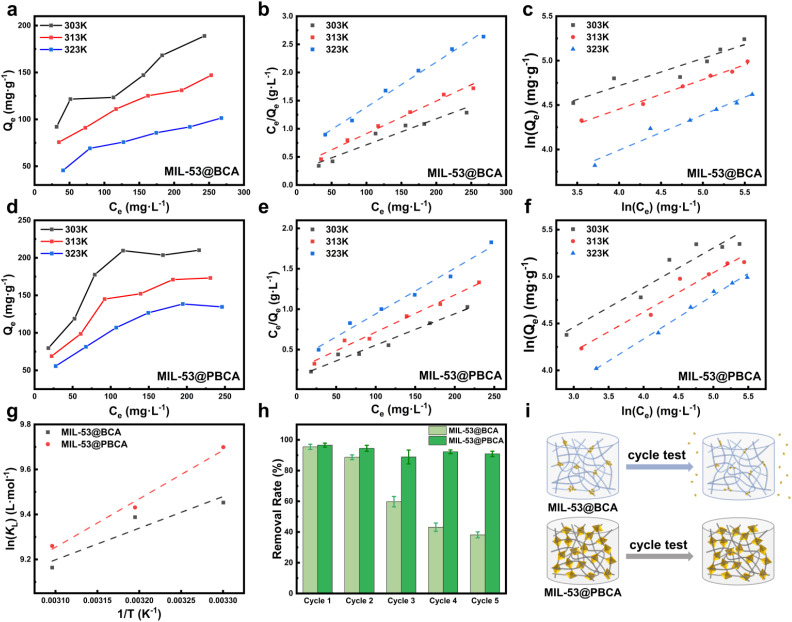
Adsorption isotherm plots at different temperatures for (a) MIL-53@BCA and (d) MIL-53@PBCA. Langmuir isotherm model of (b) MIL-53@BCA and (e) MIL-53@PBCA; Freundlich isotherm plots of (c) MIL-53@BCA and (f) MIL-53@PBCA; (g) the plots of thermodynamic parameters for the adsorption of CR onto MIL-53@BCA and MIL-53@PBCA; (h) cycle test of MIL-53@BCA and MIL-53@PBCA for CR adsorption; and (i) schematic of the probable changes in MIL-53@BCA and MIL-53@PBCA during the cycle test.

Fig. 4a–f presents a comparative isotherm analysis of CR adsorption onto MIL-53@BCA and MIL-53@PBCA composite aerogels using both the Langmuir and Freundlich models. As shown in Fig. 4a and d, the maximum equilibrium adsorption capacities at 303 K were determined to be 188.96 mg g^−1^ for MIL-53@BCA and 210.11 mg g^−1^ for MIL-53@PBCA, respectively. The fitting of the experimental data to the Langmuir model suggests that monolayer adsorption occurs on a structurally uniform surface. This observation aligns well with the microstructure of the composites, where active sites originating from the cellulose fibers, PDA coating, and MIL-53 crystals are uniformly distributed throughout the matrix.^16,39^ The saturation adsorption behavior observed in both composites supports the assumption of energetically equivalent binding sites, consistent with Langmuir's theory. At the same time, the data also exhibit good agreement with the Freundlich model, which accounts for multilayer adsorption on heterogeneous surfaces. This compatibility can be attributed to the intrinsic textural characteristics of the aerogels, such as their hierarchical porosity and surface roughness. These features create a distribution of adsorption energies across the material, allowing for multilayer dye uptake, particularly in the case of MIL-53@PBCA.^43^ It is noteworthy that MIL-53@PBCA consistently outperforms MIL-53@BCA across varying concentrations and temperatures in terms of both adsorption capacity and rate. This enhancement can be ascribed to the synergistic effects of larger and more crystalline MIL-53 domains and the increased density of functional groups provided by the PDA layer. These structural advantages likely facilitate faster dye transport and stronger dye–absorbent interactions, thus promoting more efficient adsorption kinetics and higher overall capacity.

According to the isotherm fitting results summarized in Table 2, both MIL-53@BCA and MIL-53@PBCA composite aerogels exhibited strong agreement with the Langmuir and Freundlich models, suggesting the coexistence of monolayer and multilayer adsorption mechanisms. Based on Langmuir model calculations, the maximum theoretical adsorption capacities (*Q*_max_) were determined to be 216.34 mg g^−1^ for MIL-53@BCA and 257.07 mg g^−1^ for MIL-53@PBCA, indicating the excellent affinity of both materials toward Congo Red (CR), with the PBCA-based composite showing a clear performance advantage. The Freundlich constant *n*, which reflects the strength and spontaneity of adsorption interactions, was greater than 2.0 for both materials, further confirming their favorable adsorption characteristics within the tested concentration range (50∼300 mg L^−1^). Notably, MIL-53@PBCA exhibited a higher *Q*_max_, while MIL-53@BCA demonstrated a slightly greater *n* value, implying more rapid and spontaneous initial uptake. Additionally, temperature-dependent adsorption studies revealed a decreasing trend in both the Langmuir constant *K*_L_ and the Freundlich constant *K*_F_ with increasing temperature. This thermodynamic behavior not only supports the exothermic nature of the adsorption process^44^ but also highlights that elevated temperatures may suppress adsorption efficiency for this type of system. Such insights provide valuable guidance for optimizing operational parameters in practical dye removal applications, emphasizing the importance of moderate temperatures to maintain high adsorption performance.

**Table 2:** Adsorption isotherm parameters of MIL-53@BCA and MIL-53@PBCA

	*T* (K)	Langmuir isotherm			Freundlich isotherm		
		*Q*_max_ (mg g^−1^)	*K*_L_	*R* ^2^	*K*_F_	*n*	*R* ^2^
MIL-53@BCA	303	216.45	0.0182	0.927	32.506	3.237	0.870
	313	173.01	0.0172	0.980	22.766	3.009	0.985
	323	125.00	0.0136	0.987	11.072	2.518	0.959
MIL-53@PBCA	303	257.07	0.0235	0.969	24.419	2.370	0.897
	313	217.39	0.0179	0.979	19.009	2.385	0.945
	323	176.68	0.0151	0.980	11.823	2.146	0.992

#### 3.2.4 Adsorption thermodynamics

Fig. 4g depicts the relationship between ln *K*_L_ and 1/*T* for the adsorption of CR onto MIL-53@BCA and MIL-53@PBCA, based on thermodynamic analysis. The linear fitting of experimental data using the Van't Hoff equation enabled the calculation of relevant thermodynamic parameters, as summarized in Table 3. The negative values of standard Gibbs free energy (Δ*G*°) confirm that the adsorption process is spontaneous across the tested temperature range. Additionally, the negative enthalpy change (Δ*H*°) supports the exothermic nature of the interaction, which is consistent with observations from the Langmuir-based isothermal adsorption modeling. Interestingly, the positive entropy change (Δ*S*°) suggests an increase in molecular disorder at the solid–liquid interface with increasing temperature.^45^ This entropy gain helps explain the experimentally observed decline in adsorption capacity at elevated temperatures. Mechanistically, this process likely involves the displacement of interfacial water molecules by CR dye molecules, whereby the substitution of loosely bound water by CR leads to heat release and promotes the thermal motion of both water and dye molecules. The resulting enhancement in molecular freedom contributes to the net increase in system entropy.^46,47^

**Table 3:** Adsorption thermodynamic parameters of MIL-53@BCA and MIL-53@PBCA

Adsorbate	*T* (K)	Δ*G*° (kJ mol^−1^)	Δ*H*° (kJ mol^−1^)	Δ*S*° (J mol^−1^ K^−1^)
MIL-53@BCA	303	−23.813	−11.685	40.254
	313	−24.430		
	323	−24.609		
MIL-53@PBCA	303	−24.433	−17.896	21.462
	313	−24.512		
	323	−24.867		

A comparison of the thermodynamic parameters in Table 3 reveals that both Δ*G*° and Δ*H*° values are higher for MIL-53@PBCA than for MIL-53@BCA, indicating that the PBCA-based composite exhibits greater spontaneity and stronger heat evolution during adsorption. This enhancement can be attributed to the higher MOF loading and denser distribution of active functional groups in PBCA, providing a greater number of accessible binding sites for dye uptake. Conversely, the Δ*S*° value for MIL-53@PBCA was slightly lower than that of MIL-53@BCA. This difference may be due to the distinct adsorption dynamics between the two systems. In the case of MIL-53@BCA, rapid initial adsorption likely induces more frequent interfacial interactions and displacement events, leading to greater configurational disorder at the interface. In contrast, MIL-53@PBCA undergoes a slower yet more sustained adsorption process, where dye accumulation occurs progressively, resulting in a more ordered interfacial structure and thus a relatively smaller entropy change.

#### 3.2.5 Adsorption cycle test

Fig. 4h presents the reusability performance of MIL-53@BCA and MIL-53@PBCA composite aerogels in CR removal over five consecutive adsorption–desorption cycles. The results indicate a general decline in adsorption capacity with increasing cycle numbers for both materials, although the rate and extent of degradation differ significantly. MIL-53@BCA exhibited a marked drop in adsorption efficiency after just a few cycles, suggesting poor structural stability under repeated operational conditions. This sharp decline is most likely due to the weak anchoring of MIL-53 nanoparticles on the BCA substrate. As the BCA scaffold consists of slender, one-dimensional cellulose fibers, the nanosized MIL-53 particles are only loosely attached *via* weak interfacial forces, without substantial chemical or physical embedding. Consequently, repeated adsorption and washing steps may cause extensive detachment of MIL-53 from the aerogel matrix, significantly reducing the number of available active sites. In contrast, MIL-53@PBCA demonstrated excellent cycling stability. As observed in the SEM images of MIL-53@PBCA (see Fig. 2a), the MIL-53 particles in this composite are more securely integrated within the PDA-modified fiber network. The PDA coating provides additional binding interactions and also physically encapsulates the MIL-53 crystals, thereby minimizing MOF leaching during use (see Fig. 4i). As a result, MIL-53@PBCA retained over 80% of its original adsorption capacity even after five complete regeneration cycles.

### 3.3 Photocatalytic degradation performance of organic dyes

#### 3.3.1 Photocatalytic degradation kinetics

**Fig. 5:**
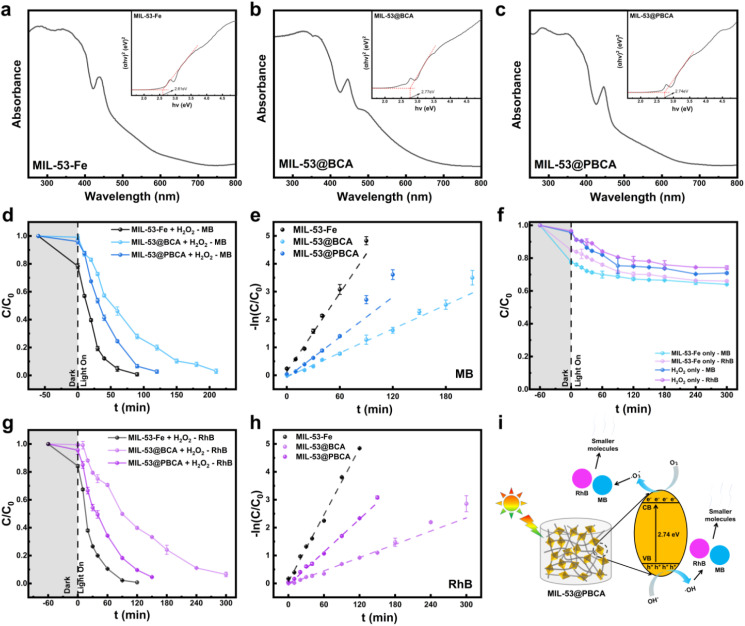
DRS spectra and band gap energies of (a) MIL-53-Fe, (b) MIL-53@BCA and (c) MIL-53@PBCA. Plots of (d) the removal rates and (e) kinetics of catalytic MB degradation by pure MIL-53-Fe, MIL-53@BCA, and MIL-53@PBCA. (f) Plots of MB/RhB removal rates by MIL-53 and H_2_O_2_ alone are shown for comparison. Plots of (g) the removal rates and (h) kinetics of catalytic RhB degradation by pure MIL-53, MIL-53@BCA, and MIL-53@PBCA. (i) Schematic representation of the mechanism for the photodegradation of MB and RhB on the surface of MIL-53 from MIL-53@PBCA under visible light irradiation.

Photocatalytic experiments demonstrated that both MIL-53@BCA and MIL-53@PBCA composite aerogels exhibited excellent degradation efficiency toward methylene blue (MB) and rhodamine B (RhB), primarily due to the Fenton-like photocatalytic activity of the embedded MIL-53-Fe component.^48^ The optical properties of pure MIL-53-Fe, MIL-53@BCA, and MIL-53@PBCA were investigated by UV-vis diffuse reflectance spectroscopy (as shown in Fig. 5a–c), and their band gap values were estimated using Tauc plots. All three samples exhibited strong visible-light absorption in the wavelength range of 250–400 nm, which is consistent with previously reported results.^48,49^ Based on the relationship *E*_g_ = 1240/*λ*, the calculated optical band gap of MIL-53-Fe was 2.61 eV (Fig. 5a). However, the band gap values of MIL-53@BCA and MIL-53@PBCA increased to 2.77 and 2.74 eV (Fig. 5b and c), respectively. This indicates that the photocatalytic activity of MIL-53-Fe decreased after compositing with BCA and PBCA, as BCA and PBCA merely serve as substrates without intrinsic photocatalytic activity. The entanglement effect of the composites may have, to some extent, attenuated the photocatalytic performance of MIL-53-Fe immobilized on the cellulose surface.

The degradation kinetics of both dyes followed a pseudo-first-order model, with correlation coefficients (*R*^2^) exceeding 0.95 for all tested systems (see Table 4), indicating a consistent decline in degradation rate over time. Under simulated visible light irradiation, MIL-53@BCA achieved 96.8% degradation of MB and 93.4% degradation of RhB after 210 and 300 minutes, respectively. In contrast, MIL-53@PBCA demonstrated superior performance, with 98.8% and 95.4% degradation of MB and RhB, respectively, within shorter reaction durations of 120 and 150 minutes. The enhanced kinetics observed in the PBCA-based system can be attributed to its higher MOF loading and more uniform particle distribution, which facilitates a greater generation of reactive oxygen species during photocatalysis. Although both composite aerogels displayed effective photocatalytic activity, their performance remained slightly inferior to that of pure MIL-53, which serves as the active photocatalyst. This reduction in activity is expected, as the cellulose and PDA components function merely as structural supports without contributing directly to photocatalytic reactivity. Nevertheless, the incorporation of these biocompatible and mechanically robust scaffolds significantly enhances the processability, stability, and reusability of the composites. Taken together, these findings confirm that MIL-53@BCA and MIL-53@PBCA not only retain the essential photocatalytic characteristics of MIL-53-Fe but also offer improved structural integrity and practical handling. Their dual functionality in both dye adsorption and visible-light-driven photocatalysis highlights their promising potential for multifunctional wastewater treatment applications.

**Table 4:** PFO model parameters for photocatalytic degradation by pure MIL-53, MIL-53@BCA, and MIL-53@PBCA

	*k*_MB_ (min^−1^)	*R* ^2^	*k*_RhB_ (min^−1^)	*R* ^2^
MIL-53@BCA	0.0143	0.958	0.0079	0.980
MIL-53@PBCA	0.0240	0.951	0.0196	0.993
Pure MIL-53	0.0469	0.970	0.0399	0.997

For anionic CR, both aerogels achieved substantial equilibrium uptake governed by combined monolayer/multilayer interactions (Section 3.2), whereas cationic MB/RhB displayed negligible dark adsorption on these scaffolds (methods). Under visible-light/H_2_O_2_, MIL-53@PBCA exhibited a higher apparent first-order rate constant than MIL-53@BCA and reached >95% dye removal within 120–150 min (Table 4), underscoring a photocatalytic pathway that outperforms any residual adsorption for MB/RhB. Mechanistically, adsorption preconcentration benefits CR removal, while for MB/RhB, the dominant contribution is derived from ROS-mediated degradation on MIL-53-Fe, with the PDA-modified scaffold facilitating higher MOF loading and more uniform active site distribution. Overall, when comparing the removal pathways of dyes by the aerogels, photocatalytic treatment exhibited markedly higher efficiency than adsorption alone.

#### 3.3.2 The effects of pH and temperature of photocatalysts on the adsorption

**Fig. 6:**
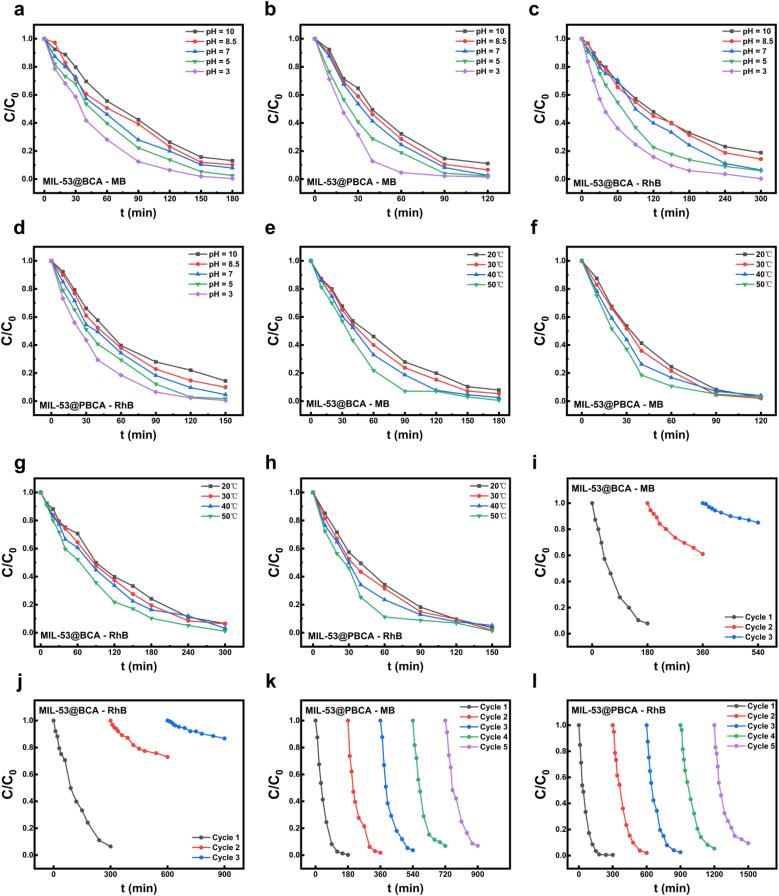
Removal rates of catalytic degradation of MIL-53@BCA and MIL-53@PBCA for MB and RhB at (a–d) different pH values (in the range of 3–10) and (e–h) temperature (in the range of 20–50 °C); (i–l) cyclic testing of the catalytic degradation of MIL-53@BCA and MIL-53@PBCA for MB and RhB.

As illustrated in Fig. 6a–h, both pH and temperature exert a significant influence on the photocatalytic performance of MIL-53@BCA and MIL-53@PBCA composite aerogels toward the degradation of methylene blue (MB) and rhodamine B (RhB). Within the pH range of 3 to 10, a clear positive correlation was observed between photocatalytic efficiency and acidity of the solution.^50^ Similarly, raising the reaction temperature from 20 °C to 50 °C resulted in a notable enhancement in dye degradation efficiency for both composites. These findings underscore the critical role of acidic conditions in stabilizing Fe^2+^/Fe^3+^ redox couples and in facilitating the generation of hydroxyl radicals (˙OH), which are central to the Fenton-like photocatalytic mechanism. Elevated temperatures further intensified the interaction between ˙OH and dye molecules and accelerated the Fe^2+^/Fe^3+^ redox cycling, thereby improving the overall kinetics of the photocatalytic process.^51–54^

#### 3.3.3 Photocatalytic degradation cycle test

As illustrated in Fig. 6i–l, the recyclability of MIL-53@BCA and MIL-53@PBCA composite aerogels in the photocatalytic degradation of methylene blue (MB) and rhodamine B (RhB) was systematically investigated. A sharp decline in the degradation efficiency of MIL-53@BCA was observed as early as the second cycle, primarily attributed to the weak interfacial adhesion between the MIL-53 particles and the bacterial cellulose matrix. The insufficient binding strength likely led to the detachment and subsequent loss of MOF components during the repeated irradiation and washing cycles. In contrast, the MIL-53@PBCA composite exhibited only a marginal decline in photocatalytic performance over five successive cycles, maintaining over 90% degradation efficiency for both dyes. This enhanced stability is likely due to the robust entanglement of MOF crystals within the dense three-dimensional fibrous network of polydopamine-coated bacterial cellulose, which effectively anchors the MIL-53 particles and minimizes their detachment, even after repeated washing and drying treatments. Nonetheless, the slight activity loss observed for MIL-53@PBCA could be attributed to the partial degradation of the MIL-53-Fe framework, likely initiated by hydroxyl radicals (˙OH) generated during the Fenton-like photocatalytic process. These reactive species may attack the organic linker (H_2_BDC) and induce localized structural collapse or leaching of active sites.^55–57^ Despite this, the overall structural integrity and functional performance of MIL-53@PBCA remained well-preserved, highlighting its superior durability and practical potential for repeated use in dye-contaminated wastewater remediation.

### 3.4 Gravity-driven oil–water separation

**Fig. 7:**
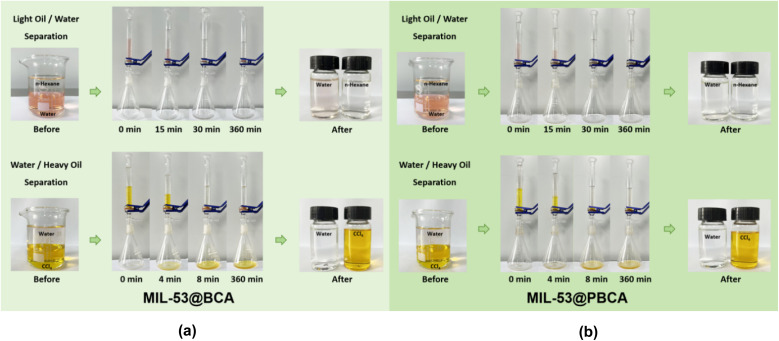
Digital images of oil/water separation by (a) MIL-53@BCA and (b) MIL-53@PBCA.

The oil–water separation performance of MIL-53@BCA and MIL-53@PBCA composite aerogels was evaluated using a gravity-driven filtration setup. After mild compression, the aerogels were inserted into the separation device to assess their efficiency across various biphasic systems, as depicted in Fig. 7. In the light oil/water system (*n*-hexane/water), where the aqueous phase was stained with 10 mg L^−1^ Congo Red (CR), the complete passage of 15 mL of water through the aerogel into the collection flask took approximately 30 minutes. Notably, both aerogels exhibited concurrent dye adsorption during this process, with MIL-53@PBCA demonstrating the complete removal of the dissolved CR, underscoring its superior affinity for anionic dyes in aqueous environments. In the heavy oil/water system (water/CCl_4_), separation occurred significantly faster and was completed within 8 minutes. This acceleration is likely due to the absence of specific interactions between the hydrophobic CCl_4_ molecules and the hydroxyl-rich cellulose matrix, enabling rapid capillary infiltration and drainage. This rapid separation process is attributed to the highly porous, interwoven fibrous network of bacterial cellulose, which facilitates rapid capillary-driven transport and reduces interfacial resistance, especially under gravitational force. Remarkably, in both systems, the upper phase was completely retained even after a prolonged filtration period of 360 minutes, demonstrating excellent phase selectivity. However, further examination revealed negligible adsorption of methyl red (MR) from the CCl_4_ phase by both MIL-53@BCA and MIL-53@PBCA, suggesting that the rapid permeation of the oil phase might preclude sufficient contact time for dye uptake. This selectivity underscores the materials' preferential interaction with hydrophilic solutes in aqueous media. These findings confirm that MIL-53@BCA and MIL-53@PBCA aerogels possess robust oil–water separation capabilities and exhibit targeted dye adsorption behavior in aqueous environments, offering new insights into their practical utility for wastewater treatment applications involving multiphase and multicomponent systems. During heavy oil/water separation, dye uptake was assessed by only a visual comparison of the lower-phase color before and after permeation (Fig. 7). Because the adsorption process is not significant and its mechanism is unclear, no spectrophotometric quantification was performed for this step; therefore, dye adsorption during separation is discussed qualitatively and not used as a performance metric.

## 4 Conclusion

In this study, bacterial cellulose aerogels (BCA) were prepared *via* the freeze-drying of membranes biosynthesized by a single strain of *Acetobacter xylinum*. Subsequent surface modification with polydopamine (PDA) yielded PDA-coated aerogels (PBCA). Through a solvothermal *in situ* growth strategy, MIL-53 crystals were successfully incorporated onto BCA and PBCA substrates, affording MIL-53@BCA and MIL-53@PBCA composite aerogels.

(1) Compared with previously reported composites based on microcrystalline cellulose, the current materials exhibit two distinct features: (i) MIL-53 crystals formed a rhombic dodecahedral morphology instead of nanorod-like structures; (ii) both MIL-53@BCA and MIL-53@PBCA possessed highly porous and interwoven fibrous networks. Due to limited growth space and weak anchoring capacity, MIL-53@BCA displayed only sparse, nanoscale MIL-53 crystals observable by SEM, whereas MIL-53@PBCA featured densely and uniformly distributed MIL-53 particles that are well integrated into the fiber matrix. The respective MOF loadings were quantified as 12.13% for MIL-53@BCA and 41.18% for MIL-53@PBCA.

(2) Both aerogels demonstrated excellent dye adsorption capacity toward Congo Red (CR). MIL-53@PBCA achieved an equilibrium adsorption of 122.17 mg g^−1^ after 24 hours, slightly higher than MIL-53@BCA, which reached saturation within 7 hours. Notably, the highly porous and fibrous structure of MIL-53@BCA enabled rapid diffusion-driven adsorption, despite its lower MOF content. Adsorption efficiency peaked near neutral pH (pH 7), and exhibited a dose-dependent trend with the amount of sorbent added. Kinetic and isotherm analyses suggested that both physical and chemical adsorption mechanisms were involved, featuring contributions from both monolayer and multilayer interactions. The thermodynamic analysis confirmed the spontaneous, exothermic nature of CR adsorption, with positive entropy change (Δ*S*°), particularly pronounced in MIL-53@BCA due to the rapid molecular rearrangement induced by fast adsorption. However, in cyclic adsorption experiments, only MIL-53@PBCA retained stable performance over multiple cycles, while MIL-53@BCA showed a sharp decline in adsorption capacity, likely due to the mechanical detachment of weakly anchored MIL-53 particles during desorption and regeneration.

(3) Regarding photocatalytic activity, both composites exhibited high degradation efficiency toward methylene blue (MB) and rhodamine B (RhB), attributed to the Fenton-like behavior of MIL-53-Fe. Their photocatalytic kinetics conformed well to first-order models. Enhanced degradation rates were observed under acidic conditions and elevated temperatures, owing to the improved generation and reactivity of hydroxyl radicals and more favorable Fe^2+^/Fe^3+^ redox cycling. Upon repeated use, MIL-53@PBCA maintained robust catalytic performance, whereas MIL-53@BCA experienced significant activity loss after the initial cycle, likely due to structural instability and partial MOF leaching under oxidative conditions.

(4) Gravity-driven oil–water separation tests confirmed that both aerogels could efficiently separate biphasic mixtures with improved rates over previous MIL-53@CA and MIL-53@PCA systems. This enhancement was mainly due to the highly porous structure of bacterial cellulose, facilitating rapid phase infiltration. Additionally, the materials demonstrated modest CR dye removal from the aqueous phase during separation, with MIL-53@PBCA showing superior adsorption. However, negligible dye removal was observed from the oil phase, possibly due to the high flow velocity of the lower liquid limiting dye–sorbent interactions. These findings highlight the dual functionality of MIL-53@PBCA in simultaneous phase separation and selective aqueous dye removal. In the heavy oil (CCl_4_/water) system, only a faint discoloration attributable to adsorption was visually discernible for MIL-53@PBCA, whereas MIL-53@BCA showed negligible change; given the rapid permeation of the lower phase, these qualitative observations were not analyzed quantitatively.

The findings of this study not only expand the functional scope of cellulose-based aerogels in advanced water purification but also contribute to the development of next-generation sustainable materials that integrate structural simplicity, ecological compatibility, and multi-functionality. By leveraging the interplay between bio-based scaffolds and crystalline porous frameworks, this work underscores a viable pathway toward a more holistic and efficient treatment of industrial effluents.

## Author contributions

Yang Chen (first Author) – conceptualization, methodology, investigation, formal analysis, data curation, visualization and writing (original draft). Shuhao Qin (corresponding author) – resources and supervision. Chengtao Gao – validation and funding acquisition. Xiao Wu – project administration and funding acquisition. Min He – funding acquisition. Daohai Zhang – resources and writing (review & editing).

## Conflicts of interest

The authors declare that they have known competing financial interest or personal relationships that could have appeared to influence the work reported in this paper.

## Data Availability

Data will be made available on request.
